# Treatment with G-CSF reduces acute myeloid leukemia blast viability in the presence of bone marrow stroma

**DOI:** 10.1186/s12935-015-0272-3

**Published:** 2015-12-21

**Authors:** Meritxell Nomdedeu, María Carmen Lara-Castillo, Amaia Etxabe, Josep María Cornet-Masana, Marta Pratcorona, Marina Díaz-Beyá, Xavier Calvo, María Rozman, Dolors Costa, Jordi Esteve, Ruth M. Risueño

**Affiliations:** Josep Carreras Leukaemia Research Institute, Campus Clínic-University of Barcelona, Rosselló 149-153, 08036 Barcelona, Spain; Department of Hematology, Hospital Clínic, Barcelona, Spain; Institut d’Investigacions Biomèdiques August Pi i Sunyer, Barcelona, Spain; Hematopathology Unit, Hospital Clínic, Barcelona, Spain; Fundació Clínic per a la Recerca Biomèdica, Barcelona, Spain

**Keywords:** G-CSF, AML, Chemotherapy priming

## Abstract

**Background:**

The resulting clinical impact of the combined use of G-CSF with chemotherapy as a chemosensitizing strategy for treatment of acute myeloid leukemia (AML) patients is still controversial. In this study, the effect of ex vivo treatment with G-CSF on AML primary blasts was studied.

**Methods:**

Peripheral blood mononuclear cells from AML patients were treated with G-CSF at increasing doses, alone or in co-culture with HS-5 stromal cells. Cell viability and surface phenotype was determined by flow cytometry 72 h after treatment. For clonogenicity assays, AML primary samples were treated for 18 h with G-CSF at increasing concentrations and cultured in methyl-cellulose for 14 days. Colonies were counted based on cellularity and morphology criteria.

**Results:**

The presence of G-CSF reduced the overall viability of AML cells co-cultured with bone marrow stroma; whereas, in absence of stroma, a negligible effect was observed. Moreover, clonogenic capacity of AML cells was significantly reduced upon treatment with G-CSF. Interestingly, reduction in the AML clonogenic capacity correlated with the sensitivity to chemotherapy observed in vivo.

**Conclusions:**

These ex vivo results would provide a biological basis to data available from studies showing a clinical benefit with the use of G-CSF as a priming agent in patients with a chemosensitive AML and would support implementation of further studies exploring new strategies of chemotherapy priming in AML.

**Electronic supplementary material:**

The online version of this article (doi:10.1186/s12935-015-0272-3) contains supplementary material, which is available to authorized users.

## Background

According to the hierarchic model of cancer, AML cells are maintained by a subset of cells, called leukemic stem cells (LSCs), which have the capacity of self-renewal and differentiation [1]. Due to their stem cell–like properties, LSCs are the cell population showing a highest resistance to conventional chemotherapeutic agents used in AML treatment, such as anthracyclines and cytarabine [2]. Additionally, chemotherapy resistance may be partially explained by the protective effect exerted by bone marrow niche on leukemia cells against virtually any type of therapy [3], and might contribute to the high incidence of relapse observed after frontline chemotherapy [2]. Therefore, AML therapy requires the complete eradication of LSCs in order to achieve long-term cure. Administration of granulocyte colony-stimulating factor (G-CSF) concurrently with induction chemotherapy, as a priming strategy, has been used based on pre-clinical data suggesting a sensitization of LSCs to the cytotoxic effect of conventional chemotherapy with the concomitant administration of G-CSF via differentiation induction, cell cycle entry stimulation, and mobilization from the bone marrow [4]. Additionally, G-CSF could exert its anti-leukemic effect inducing mobilization out of the protective bone marrow microenvironment by disrupting the CXCR4-CXCL12 axis [5].

In clinics, the simultaneous administration of G-CSF and chemotherapy as a priming strategy has yielded conflicting results. Thus, some reports have described a favorable effect of G-CSF priming in favorable and the intermediate risk AML patients, without clinical benefit in patients with unfavorable cytogenetics [6, 7].In contrast, other studies have failed to show a clinical effect of priming strategies, although these conflicting results must be due in part to different patient inclusion criteria, disease status and treatment administered [8, 9].

G-CSF is the main cytokine that drives granulopoiesis exerting its function through the G-CSF receptor (G-CSFR). G-CSFR is a single transmembrane receptor that belongs to the cytokine receptor type I superfamily [10]. The intracellular region lacks intrinsic tyrosine kinase activity, but contains two conserved membrane-proximal motifs: box 1 and box 2, involved in Jak kinase activation. In the membrane-distal intracellular tail, there is a more distal box 3 motif and specific tyrosine residues important for signaling transduction [11]. Upon ligand recognition, G-CSFR homodimerizes allowing trans-phosphorylation and activation of Jak2 kinases that are constantly bound to box 1 and 2, which consequently initiates downstream intracellular signaling cascades, including Jak/STAT/Socs, Ras/Raf/Erk and PI3K/Akt pathways. As a result, transcription changes that impact on survival, migration, proliferation and differentiation are induced [12]. G-CSF/G-CSFR axis regulates myelopoiesis under basal conditions of hematopoiesis and neutrophil production during emergency granulopoiesis [4]. G-CSF signaling is implicated in hematopoietic stem/progenitor cell mobilization through three different mechanisms G-CSFR-independent: induction of proteases, attenuation of function of adhesion molecules, and disruption of signaling initiated by CXCL12 (SDF-1) through CXCR4 [13]. G-CSF also promotes mobilization of mature myeloid cells via induction of the transcriptional repressor GFI-1, which attenuates their responsibleness to bone marrow CXCL12 [14].

Here, we analyzed the ex vivo effect of G-CSF on primary AML samples in order to elucidate the biological mechanisms underlying chemotherapy priming strategies with this agent [15–18]. Our results suggest that the anti-leukemic effect of G-CSF treatment is mostly stroma-dependent. Moreover, cell viability and clonogenic capacity was significantly reduced upon G-CSF treatment in chemosensitive AML samples. Thus, this correlation between pre-clinical ex vivo observation and clinical results might be used to anticipate clinical response to chemotherapy and select optimal therapy.

## Results

**Fig. 1:**
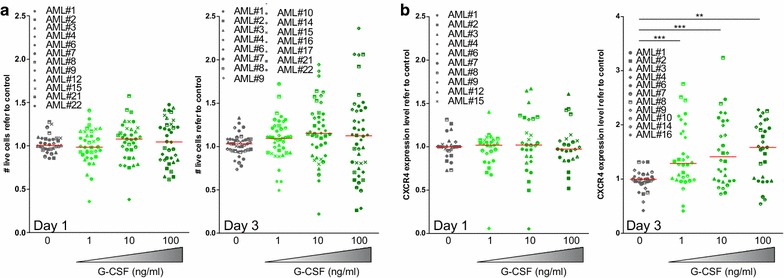
G-CSF treatment spares AML blasts while it increased surface CXCR4 expression. Primary patient AML cells were cultured in the presence of 0.1, 1 and 10 μg/mL of G-CSF for 24 and 72 h. **a** Cell viability was measured by live-death discrimination (7-AAD) and volumetric count by flow cytometry. **b** CXCR4 surface expression was measured by flow cytometry. ** p < 0.01; *** p < 0.005

**Fig. 2:**
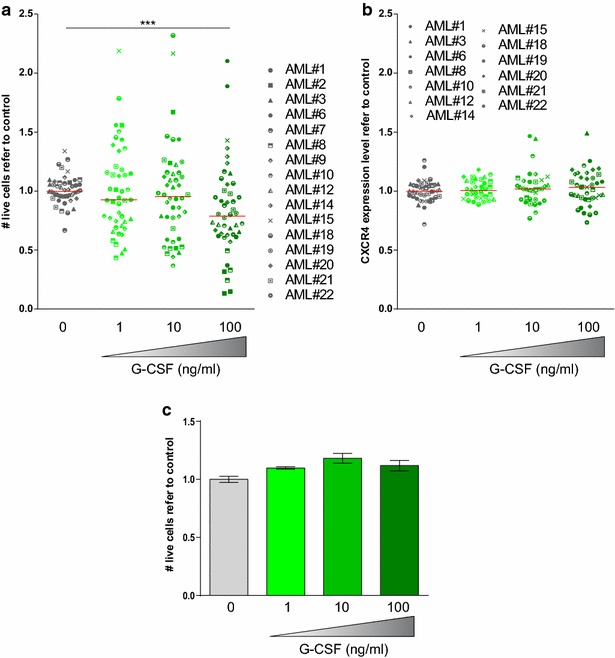
G-CSF treatment significantly reduced cell viability of AML blasts in the presence of bone marrow stroma, while CXCR4 expression remained unaffected. Primary patient AML cells were co-cultured with HS-5 human bone marrow stromal cell line and treated with G-CSF at increasing concentrations for 24 h. **a** Cell viability was measured by live-death discrimination (7-AAD) and volumetric count by flow cytometry. **b** CXCR4 surface expression was measured by flow cytometry. **c** HS-5 human bone marrow stromal cells were treated with G-CSF at increasing concentrations for 24 h. Cell viability was measured as for **a**. *** p < 0.005

In order to study the effect of G-CSF on cell viability, bulk AML blast population was treated with increasing doses of G-CSF. In concordance with previous data [19], no significant differences were observed in cell number 24 and 72 h after treatment (Fig. 1a, Additional file 1: Figure S1A). Treatment with G-CSF upregulated CXCR4 expression in a dose-dependent fashion (Fig. 1b, Additional file 1: Figure S1B), demonstrating that although G-CSF signaling was activated, no influence over cell viability was observed. In contrast, when bone marrow stroma cells were added to mimic their physiological niche, G-CSF treatment induced a significant reduction in the overall cell viability at 24 h (Fig. 2a, Additional file 1: Figure S1C). Interestingly, no changes in CXCR4 expression were observed (Fig. 2b, Additional file 1: Figure S1D). The reduction of cell viability observed on AML cells treated with G-CSF was not due to cytotoxicity on bone marrow stroma cells. In fact, stroma cells remained unaffected upon G-CSF treatment in terms of cytotoxicity or morphology (Fig. 2c).

**Fig. 3:**
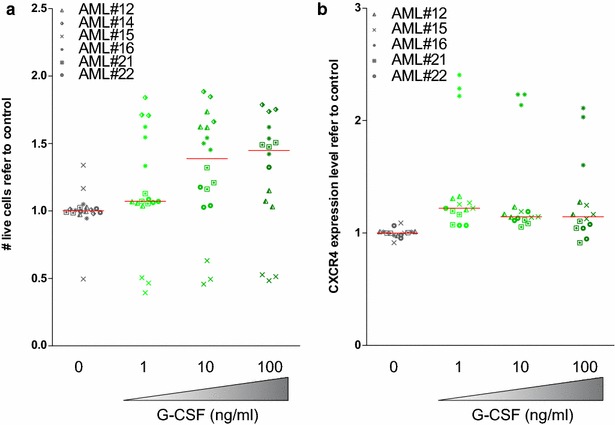
Cell viability of the AML blast population and CXCR4 expression remained unaffected when no direct contact with stroma cells was allowed. Primary patient AML samples were co-cultured with HS-5 cells in a *transwell* system and treated with G-CSF at increasing concentrations for 24 h. **a** Cell viability and **b** CXCR4 surface expression were measured by flow cytometry

After, the requirement of a direct contact with stroma cells was investigated. Cell viability of the AML blast population remained unaffected when no direct contact with stroma cells was allowed by using a *transwell* system (Fig. 3a). In addition, CXCR4 expression remained unaffected (Fig. 3b). These findings suggest that the cytotoxic effect of G-CSF on the AML blast population requires not only the presence of bone marrow microenvironment cells, but also a direct contact with stroma cells. However, taking into account the absence of changes in the CXCR4 levels, it is unlikely that the effect is mediated by the CXCR4/CXCL12 axis. In fact, G-CSF exerts its effect also on BM stroma cells.

**Fig. 4:**
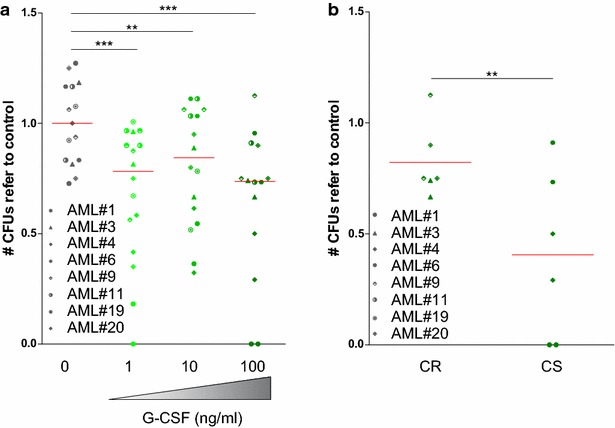
G-CSF treatment reduced the clonogenic capacity of AML bulk population. Primary patient AML samples were treated with G-CSF as indicated for 18 h. **a** CFU-B were counted based on morphological criteria. **b** Relative change in the clonogenic capacity after G-CSF treatment in patients who achieved a complete response after induction treatment (CS) compared to primary chemorefractory patients (CR). Each *symbol* corresponds to an AML patient. All data was normalized against control. *p < 0.05, **p < 0.01, ***p < 0.001

The ability of G-CSF to induce differentiation and, eventually, sensitization of LSCs to chemotherapy may be relevant in a clinical setting to enhance anti-leukemic effect of current standard treatment [4]. Since a clonogenic capacity assay in a semi-solid media remains the gold standard technique to study the LSC function ex vivo, primary AML samples were treated with G-CSF for 18 h and a significant reduction of CFU-B colonies was observed in a dose-dependent manner upon treatment with G-CSF (Fig. 4a), suggesting that G-CSF exerts its effect at least partially on LSC function.

The beneficial effect of the concomitant use of G-CSF with induction chemotherapy reported in previous clinical studies was mainly observed in the subset of patients with a cytogenetic intermediate-risk AML [20]. Thus, the response to G-CSF treatment ex vivo of primary AML cells was compared to the observed clinical patient response to the induction chemotherapy regimen. No differences in cell viability were detected both, in the presence or absence of stroma cells, depending on clinical response to induction treatment. In contrast, the clonogenic capacity was markedly reduced in samples from patients who achieved a first complete remission after induction treatment, compared to samples from chemorefractory patients (Fig. 4b).

## Discussion

As a summary, G-CSF reduced the viability of leukemic cells in the presence of stroma cells, highlighting the importance of the microenvironment for the anti-leukemia effect of G-CSF. Interestingly, G-CSF treatment decreased the clonogenic capacity of AML samples. As the clonogenic assay remains the gold standard for assessing LSC functionality, this finding suggests that G-CSF exerts its effect at least partially on LSC. It is in contrast with the lack of effect of G-CSF on leukemic cell viability in the absence of stroma. However, as the frequency of LCS inside the AML bulk population is relatively low, the effect of G-CSF in this leukemic population could be challenging to be measured by flow cytometry. The observed correlation between the degree of reduction of clonogenic capacity of primary AML cells following exposition to G-CSF and clinical response observed in the same patients to induction chemotherapy provide insights to the selective benefit in determined cytogenetic patient populations reported in previous clinical studies. These results are consistent with the previously reported data by the HOVON cooperative group [6], where the unfavorable cytogenetic risk group of patients did not obtain a major clinic benefit from G-CSF priming strategy. The fact that AML samples from primary refractory patients showed a lower reduction in their clonogenic capacity after treatment with G-CSF supports the notion that the priming strategy with this agent should be more efficient in patients with a greater degree of chemosensitivity. Therefore, ex vivo clonogenicity assays could be used to predict clinical response to priming strategies, although further studies aimed to analyze this correlation between experimental and clinical results are required.

## Conclusions

1. Reveals that the cytotoxic effect of G-CSF treatment on AML is stroma-dependent.
2. Demonstrates that the presence of G-CSF reduces the clonogenic capacity of AML blasts, especially in chemosensitive AML cells.
3. Identifies G-CSF as a priming agent in primary chemosensitive patients and supports further studies to explore new strategies of chemotherapy priming in AML patients.
4. Establishes a biological explanation to clinical studies, resolving the discrepancies in the field regarding G-CSF treatment.

## Methods

### Patient samples

Primary AML samples were obtained from patients diagnosed with AML at Hospital Clínic of Barcelona before receiving any treatment. Diagnoses were based on WHO 2008 criteria and their main characteristics are summarized in Table 1. All patients provided written informed consent in accordance with the Declaration of Helsinki, and the study was approved by the Ethics Committee of Hospital Clínic of Barcelona.

**Table 1:** AML patients’ characteristics

AML sample	Gender	Age (yo)	WHO 2008 category	Karyotype	NPM1,FLT3-ITD, CEBPA and DNMT3A mutational status	Chemosensitivity
#1	M	28	AML NOS, without maturation	46, XY	FLT3 ITD	CS
#2	M	40	AML with mutated CEBPA	46, XY	CEBPA^mut^	
#3	F	34	AML with myelodysplasia-related changes	45, XX, −7	FLT3 ITD^neg^, NPM1^wt^	CR
#4	M	45	AML with t(6,9)(p23;q34);*DEK*-*NUP214*	46, XY, t(6;9)(p23;q34)	FLT3 ITD	CS
#6	M	61	AML with t(8;21)(q22;q22); *RUNX1*-*RUNX1T1*	45, X–Y, t(8;21)(q22;q22)[19]/46, XY	FLT3 ITD^neg^, NPM1^wt^	CS
#7	F	58	AML with myelodysplasia-related changes	46, XX, del(5)(q23q33), t(8;9)(p11;q34)	FLT3 ITD^neg^, NPM1^wt^	
#8	M	24	AML with myelodysplasia-related changes	46, XY	FLT3 ITD^neg^, NPM1^wt^	
#9	M	49	AML with myelodysplasia-related changes	Complex karyotype	FLT3 ITD^neg^, NPM1^wt^	
#10	F	66	AML with myelodysplasia-related changes	46, XX, del(11)(q22q23)	FLT3 ITD	
#11	M	22	AML with t(8;21)(q22;q22); *RUNX1*-*RUNX1T1*	45, X, −Y, t(8;21)(q22;q22)/46, XY	FLT3 ITD	CS
#12	F	22	AML with inv(16)(p13.1q22);CBFB-MYH11	46, XX, inv(16)(p13q22)/46, XX	FLT3 ITD^neg^, NPM1^wt^	
#13	M	37	AML with mutated NPM1	46, XY	NPM1^mut^, DNMT3A^mut^	
#14	M	42	AML with mutated NPM1	46, XY	NPM1^mut^, FLT3 ITD	
#15	F	60	AML with myelodysplasia-related changes	48, XX, +8, +21	FLT3 ITD^neg^, NPM1^wt^	
#16	F	62	AML NOS, with maturation	46, XX	FLT3 ITD^neg^, NPM1^wt^	
#17	F	60	AML with myelodysplasia-related changes	Complex karyotype	FLT3 ITD^neg^, NPM1^wt^	
#18	F	55	AML with inv(16)(p13.1q22);CBFB-MYH11	46, XX, inv(16)(p13q22)/46, XX	FLT3 ITD^neg^, NPM1^wt^	
#19	M	63	AML with myelodysplasia-related changes	46, XY	FLT3 ITD	CR
#20	M	61	AML with mutated NPM1	46, XY	NPM1^mut^, DNMT3A^mut^	CR
#21	F	51	AML NOS, acute monoblastic leukemia	Not available	NPM1^mut^, FLT3 ITD^neg^	
#22	F	36	AML with mutated NPM1	46, XX	NPM1^mut^, FLT3 ITD^neg^	

*M* male, *F* female, *yo* years old, *FLT3-ITD* FLT3 internal tandem duplication, *CEBPA* ^*mut*^ biallelic CEBPA mutation, *FLT3 ITD* ^*neg*^ absence of FLT3-ITD, *NPM1wt* wild-type NPM1, *NPM1* ^*mut*^ mutated NPM1, *Wt* wildtype

### Cell viability assay

Five-hundred cells per ml were cultured in 96-well plates in complete IMDM medium (PAA). G-CSF (PrepoTech) was added at different concentrations. Co-culture experiments with stromal cells were performed seeding 30 × 10^3^ HS-5 cells together with 1 × 10^5^ AML cells in a 96-well plate. Cell viability was measured by 7-AAD (eBioscience) exclusion and cell count was obtained by volume in a FACSCanto II cytometer (BD). In co-culture experiments, AML cells were discriminated based on their CD45 expression (BD). For CXCR4 determination, cells were stained on the surface with an anti-CXCR4 antibody (clone 12G5, BD) and samples were acquired in a FACSCantoII (BD). Flow cytometry fcs files were analyzed using FlowJo software (TriStar) and statistics were calculated in GraphPad (Prism software). Each experimental point was normalized against the mean value corresponding to the vehicle treated control triplicates. A 2-tailed Mann–Whitney test was performed to determinate statistical significance.

### Transwell assay

Membranes with 1 μm pore size (Falcon) were used in a 24-wells format. A total of 100,000 HS-5 cells were added to the lower compartment. 300,000 primary AML patient samples were added to the upper compartment. Both compartments were filled with complete media as described above. Cells were incubated 24 h and analyzed as for viability assays.

### CFU assay

AML primary samples were treated for 18 h with G-CSF (PrepoTech) at different concentrations in complete IMDM medium and cultured in MethoCult H4034 (StemCell Technologies) for 14 days. Colonies were counted based on cellularity and morphology criteria.

## Additional file

10.1186/s12935-015-0272-3 G-CSF treatment significantly reduced cell viability of AML blasts in the presence of bone marrow stroma. Primary patient AML cells were cultured in the presence of 0.1, 1 and 10 μg/mL of G-CSF for 24 and 72 h. (**A**) Cell viability was measured by live-death discrimination (7-AAD) and volumetric count by flow cytometry. (**B**) CXCR4 surface expression was measured by flow cytometry. The same primary patient AML sample set was co-cultured with HS-5 human bone marrow stromal cell line and treated with G-CSF at increasing concentrations for 24 h. (**C**) Cell viability was measured by live-death discrimination (7-AAD) and volumetric count by flow cytometry. (**D**) CXCR4 surface expression was measured by flow cytometry.
