# Supplementary figures and images for: Treatment with G-CSF reduces acute myeloid leukemia blast viability in the presence of bone marrow stroma
Source: Cancer Cell Int. 2015 Dec 21;15:122. doi: 10.1186/s12935-015-0272-3 (PMC4687155; doi:10.1186/s12935-015-0272-3)

Nomdedeu et al. Supplementary Figure 1

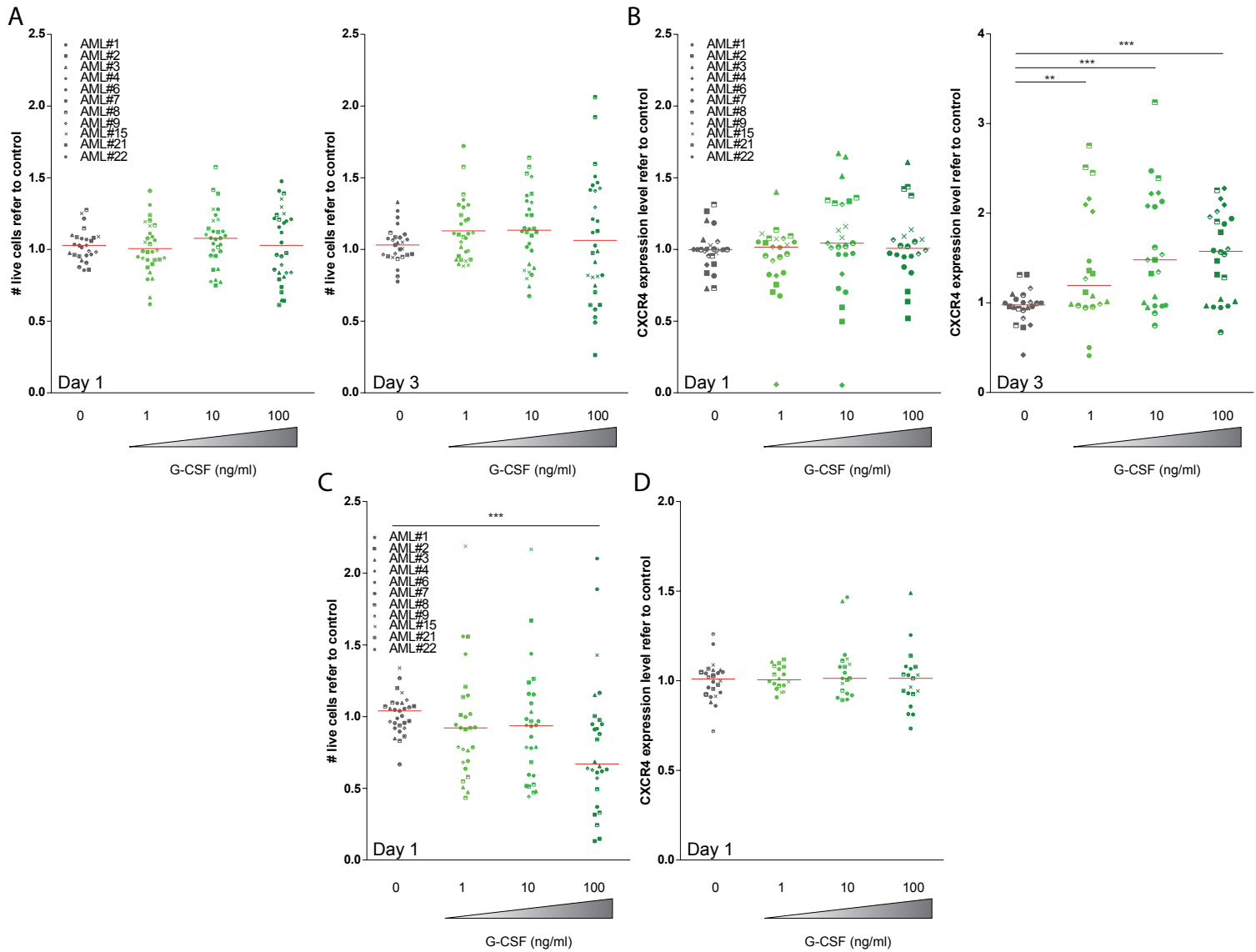

Supplement: Supplementary file 1 — 10.1186/s12935-015-0272-3 G-CSF treatment significantly reduced cell viability of AML blasts in the presence of bone marrow stroma. Primary patient AML cells were cultured in the presence of 0.1, 1 and 10 μg/mL of G-CSF for 24 and 72 h. (A) Cell viability was measured by live-death discrimination (7-AAD) and volumetric count by flow cytometry. (B) CXCR4 surface expression was measured by flow cytometry. The same primary patient AML sample set was co-cultured with HS-5 human bone marrow stromal cell line and treated with G-CSF at increasing concentrations for 24 h. (C) Cell viability was measured by live-death discrimination (7-AAD) and volumetric count by flow cytometry. (D) CXCR4 surface expression was measured by flow cytometry. [file 12935_2015_272_MOESM1_ESM.pdf]
